# Working together: gut microbe-microbe interactions shape host inflammation

**DOI:** 10.1128/iai.00512-24

**Published:** 2025-06-13

**Authors:** Ally Lawing, Rachel Bleich

**Affiliations:** 1 Department of Biology, Appalachian State University 592748, Boone, North Carolina, USA; University of Pittsburgh, Pittsburgh, Pennsylvania, USA

**Keywords:** microbe-microbe interactions, polymicrobial interactions, inflammation

## Abstract

Inflammatory bowel disease (IBD) is a debilitating disorder characterized by chronic intestinal inflammation that currently has no cure. Alterations to the composition of the gut microbiota, including reduced microbial diversity and expansion of pathobionts like Enterobacteriaceae, are implicated in IBD. While this dysbiosis has been well-documented, our understanding of the function of these microbes in the development and progression of IBD is more limited. As part of the gut microbiota, these microbes undergo complex interactions with many other microorganisms that impact the structure and function of the microbial community and the health of the host. These include competitive interactions for nutrients and space and cooperative interactions that help optimize resource utilization and microbial fitness. In this minireview, we discuss the microbe-microbe interactions that can impact host inflammation and IBD progression and treatment. Due to their association with IBD, we put special emphasis on interactions between Enterobacteriaceae and other members of the microbiota that are competitive, commensal, and mutualistic. To better understand these interactions, the signals that mediate microbial interactions are highlighted, including contact-dependent and contact-independent mechanisms. Finally, mucosal biofilms involving pathobionts are examined due to their proximity to the host and ability to influence inflammation.

## INTRODUCTION

Inflammatory bowel disease (IBD) is a debilitating disorder that causes chronic inflammation of the digestive tract and currently affects over 2 million Americans (1, 2). With diagnoses that occur frequently before the age of 35, this creates a lifelong battle that impacts quality of life along with physical, psychological, and financial burdens. IBD includes Crohn’s disease (CD), a chronic inflammatory condition of the entire gastrointestinal (GI) tract affecting more than 700,000 people in the United States, and ulcerative colitis (UC), a chronic inflammatory condition of the large intestine affecting approximately 0.4% of people in North America (3, 4). IBD currently has no cure, and patients with IBD are at a greater risk of developing comorbidities like stricture formation or inflammation-associated colorectal cancer (5–7). The etiology of IBD is unclear but is influenced by a combination of environmental and genetic factors (8, 9). Many genes linked to IBD risk are connected to pathways involved in immune-microbe interactions, including microbial detection, epithelial barrier function, and immune activation (8, 10).

Commensal microbes (microbiota) in the human GI tract are implicated in IBD and its comorbidities, and alterations to the composition of the IBD microbiota have been well-documented, including an increase of mucosal bacteria (11–18). Decades of research have examined differences in the composition of the microbiota in healthy individuals and patients with IBD and documented a loss of microbial diversity and expansion of potential pathogens associated with IBD (12–15, 17, 19, 20). The phylum Bacillota, which contains many protective anaerobes, is often reduced in the stool of IBD patients, while members of the phylum Pseudomonadota are commonly increased in those with IBD (12, 21, 22). There are many of these observed correlations between inflammation status and altered microbiome composition, a community structure generally termed “dysbiosis” (19, 20). Enterobacteriaceae, a family within Pseudomonadota, including strains of adherent-invasive *Escherichia coli* (AIEC), have been highly associated with IBD (17, 23–25). AIEC cannot be discriminated from other *E. coli* by genomic features, such as simple screening for the presence of a marker gene from patient microbiomes, but are distinguished functionally by *in vitro* assays (17, 22, 26). These assays include the ability to adhere to and invade intestinal epithelial cells and to survive and replicate in macrophages (7, 17).

Although alterations to the composition and diversity of the microbiota during IBD have been well-documented, direct links to specific pathobionts are not as prevalent (27). Most of the known associations between microbes and inflammation have been characterized through sequencing to monitor changes in microbial abundance compared to healthy controls or by mono-colonizations to directly study host-microbe interactions in various germ-free models. However, little is known regarding the microbe-microbe interactions within the gut that impact this dysbiosis and help shape microbiota alterations during inflammation. Additionally, it has become apparent that beyond taxonomic changes and dysbiosis, functional alterations to the microbiota are critical to the impact of the microbiota on inflammation and host health and should be a key consideration in future research (28).

Gut bacteria live in a complex community where they interact with many other microorganisms. Microbial interactions are important for determining how these bacteria will function within the gut environment (29–31). The signals bacteria receive from other microbes influence which genes they transcribe, the proteins and small molecules they produce, and the phenotypes they display. However, we have relatively little understanding of the mechanisms behind these interactions and how they ultimately impact the structure and function of the microbial community and the health of the host. Additionally, it is difficult to determine if microbes are actually interacting within the complexity and sheer volume of the gut microbiota. More work understanding the spatial organization of the microbiota could help better define interacting bacterial partners important during inflammation.

In this minireview, we examine the microbe-microbe interactions that can impact host inflammation and IBD progression and treatment. Due to their association with IBD, we put special emphasis on interactions between Enterobacteriaceae and other members of the microbiota that are both competitive and cooperative. Biofilms on the gut mucosa have been linked to IBD, with mucosal bacteria found at higher density in patients with IBD (32–34). AIEC is known to form biofilms on the mucosa of patients with IBD and is a stronger biofilm producer than non-AIEC strains (35, 36). Thus, we also focus on the important interactions that happen in mucosal biofilms.

## PRO-INFLAMMATORY BACTERIA

The gut microbiome is a complex ecosystem comprised of a diverse array of microorganisms. It plays a crucial role in fostering a beneficial and synergistic relationship with the host by regulating immune responses, maintaining homeostasis, and supporting metabolic processes (37). However, pro-inflammatory bacteria emerge and expand in the gut microbiota of patients with IBD as part of dysbiosis within the gut microbiome (38). These pathobionts are characterized by their capacity to interact with the host to elicit an inflammatory response (39). Numerous bacterial strains contribute to this pro-inflammatory response and colonize GI tract regions. Research has identified certain strains of *E. coli*, particularly AIEC, and *Enterococcus* that have an increased abundance and display pro-inflammatory mechanisms along with *Clostridium* and *Ruminococcus* in the microbiota of individuals with IBD when compared to healthy individuals (40).

Pathobionts can profoundly contribute to inflammation in IBD through various mechanisms. Enhanced levels of pro-inflammatory bacteria can hinder the colonization of beneficial bacterial species, such as those from Bacteroidota and Bacillota phyla, leading to dysbiosis (41). This dysbiosis increases intestinal barrier permeability, triggering an immune response with increased bacterial translocation out of the GI tract (42). As luminal and translocating bacteria proliferate, they release antigens and activate toll-like receptors (TLRs) essential in mediating inflammatory responses (43). For instance, the direct upregulation of TLR4 and myeloid differentiation factor 2 via cell surface molecules, such as lipopolysaccharide (LPS), can drive inflammatory pathways in *Campylobacter concisus* (44). These pro-inflammatory microbes release a range of inflammatory cytokines—including tumor necrosis factor-alpha (TNF-α), interferon-gamma, interleukin-1 beta (IL-1β), IL-6, IL-17, and IL-23—which are carried into the bloodstream by macrophages and disseminated throughout the body (45). Additionally, these bacteria can utilize various metabolites to induce inflammation through multiple pathways including the degradation of short-chain fatty acids (SCFAs), the downregulation of secondary bile acid production—which subsequently affects Th17 cell function—and the increased production of metabolites such as histamine and hydrogen sulfide, which compromise the intestinal epithelial barrier’s integrity (46). Together, these mechanisms create a continuous cycle of dysbiosis, immune activation, and barrier dysfunction, ultimately driving chronic inflammation and tissue damage in IBD. Understanding these interactions is crucial for developing effective therapeutic strategies to restore gut homeostasis and reduce disease progression.

## MICROBE-MICROBE INTERACTIONS

Both positive and negative microbial interactions help maintain a stable gut microbial community and influence host health (47, 48). Negative interactions like competition exclude or suppress some microbes from nutrients and space in the gut, while positive interactions can help optimize resource utilization and microbial fitness (47). Some research suggests that negative interactions dominate the microbial interactions in the gut (49, 50). However, positive interactions with one or both microbes have been more recently widely reported for the role they play in the shaping of microbial communities (51–54). These positive interactions include unidirectional commensalism and bidirectional mutualism (47, 48). While great strides have been made to uncover mechanisms of microbe-microbe interactions, more work is needed to tease apart the breadth of interactions occurring within the inflamed gut microbiome.

### Competition

Microbes within the gut compete for nutrients and space in many ways including Type VI and Type VII secretion systems (T6SS and T7SS), bacteriocins, and indirect competition for shared resources (47, 55). T6SSs are widely distributed across gram-negative bacteria and mediate contact-dependent cell-cell interactions with bacterial and eukaryotic hosts (47, 56). T6SSs are protein nanomachines that are used to deliver an array of effector proteins directly into target cells primarily for interbacterial competition (47, 56). Within the gut microbiome, T6SSs have been found in Bacteroidota and Pseudomonadota, including some examples of AIEC (47, 57, 58). A mouse gut commensal *E. coli* has been reported to outcompete pathogenic *Citrobacter rodentium* using two T6SSs, indicating its utility by resident microbiota to compete in the gut environment (59).

Bacteriocins are ribosomally synthesized and post-translationally modified peptides with antimicrobial properties and are produced and secreted from bacterial cells (60, 61). Members of the Enterobacteriaceae family can produce microcins, a class of short peptide bacteriocins, that are secreted during nutrient depletion and have antimicrobial activity against related strains of bacteria (61, 62). Microcins have been identified in commensal strains of *E. coli* including the probiotic strain Nissle 1917 that can compete with commensal *E. coli*, AIEC, and *Salmonella enterica* in a microcin-dependent manner during intestinal inflammation (63). During inflammation, access to iron becomes more limited for gut microbes, and microbes rely on siderophores to scavenge for iron (64). This increases microcin expression induced by iron deprivation and microcin-mediated competition that only occurs under iron-limited conditions (61, 63). Some microcins are linked to siderophores (siderophore-microcins), allowing them to enter bacteria by mimicking iron-siderophore complexes (65). These siderophore-microcins have been identified in intestinal strains of *Klebsiella* and *E. coli* (65). More work is needed to study the full implications of these microcins in IBD and their potential utility in modulating the microbiome for IBD treatment.

There are additional biosynthetic pathways that produce small molecules and peptides encoded within the gut microbiome that could influence microbe-microbe interactions. These types of molecules are encoded within biosynthetic gene clusters (BGCs) in bacterial genomes, making these biosynthetic pathways easy to identify (66). Colibactin is a genotoxin produced by a nonribosomal peptide synthetase-polyketide synthase BGC by several different bacterial members of the gut microbiota, but mainly in B2 phylotype *E. coli* and other Enterobacteriaceae (67, 68). Although it has been well-studied for eliciting host DNA damage and is implicated in colorectal cancer, less is known about its role in microbe-microbe interactions (7, 24, 55, 69–72). Previous studies indicate colibactin production may cause a shift to the gut microbiota in mice and inhibit the growth of some *Staphylococci* through DNA damage (73–75). An alternative mechanism for colibactin’s role in microbial interactions was recently described where colibactin producers could target other bacteria by activating prophages through the bacterial SOS response (67, 74). Prophages were activated across diverse bacteria, suggesting a role for colibactin in shaping the composition of the microbiota (67). Another recent study combined a computational algorithm and synthetic biology tools to uncover 13 BGCs that encode Type II polyketides within human microbiome metagenomic data and showed that two of the discovered products have strong antibacterial activity (66). This suggests there are potential BGC products to be uncovered that may be important for mediating microbe-microbe competition.

During IBD, certain microbes like Enterobacteriaceae will utilize changes in available nutrients due to the altered environment of the gut to outcompete and better colonize, particularly at the mucosal niche. For example, during inflammation, it has been well-documented that increased oxygen at the epithelium fuels the expansion of facultative anaerobes that can do aerobic respiration (76–79). Depletion of butyrate-producing microbes in the gut reduces signaling through the butyrate sensor peroxisome proliferator-activated receptor gamma, which increases bioavailable oxygen at the epithelium by driving colonocytes toward β-oxidation (80, 81). Additionally, Enterobacteriaceae can also utilize epithelial-derived nitrate for anaerobic nitrate respiration (79, 80, 82). Along with oxygen, this contributes to their expansion during mouse models of colitis (82, 83).

In addition to utilizing oxygen and nitrate at the mucosa, Enterobacteriaceae also have the metabolic flexibility to utilize an array of carbon sources that allow them to persist in the gut and promote inflammation (55, 84). Carbohydrates in the intestinal mucus are important energy sources for gut microbes like AIEC. The fermentation of fucose produces 1,2-propanediol, which is metabolized by *E. coli* that use propanediol dehydratase (PduC) to convert 1,2-propanediol to propionate and eventually pyruvate for use in the tricarboxylic acid cycle (85, 86). PduC is enriched in the genomes of AIEC relative to nonpathogenic *E. coli* and helps drive AIEC-induced intestinal T cell inflammation (87, 88). AIEC can also utilize ethanolamine, which is readily accessible during intestinal inflammation, as both a carbon and nitrogen source (84, 89). Bile salts, which are common metabolites in the gut, induce expression of secondary metabolism pathways allowing AIEC to utilize ethanolamine (85). Intestinal inflammation can also reprogram AIEC strains like LF82 to catabolize the amino acid l-serine to improve growth and outcompete other strains in the inflamed gut (90). Previous research shows increased expression of pathways for amino acid metabolism generally in AIEC during inflammation (90). Thus, understanding how the changing environment and nutrient composition of the gut during inflammation influence microbial interactions is an important avenue to keep exploring.

### Positive interactions

Microbes within the gut microbiota can have positive interactions with one another to help improve bacterial growth and shape the microbial community (47). However, in the context of disease, these interactions can increase virulence and be detrimental to the host. Many of these positive interactions involve complementary metabolism or cross-feeding between the microbes interacting. These interactions can be commensal or mutualistic and include catabolic and anabolic processes such as complementary biosynthetic capabilities, exchange of beneficial metabolites, or polymer degradation (47, 48, 51, 91).

Commensalism is a unidirectional interaction where one species benefits without causing harm to or benefiting the other species and has been suggested to be the most common positive interaction in bacterial communities (47, 52). Most of the established commensal microbe-microbe interactions in the gut involve species of *Bacteroides* and *Bifidobacterium* that can utilize a wide variety of carbohydrates and release degradation products, benefitting other members of the microbiota (92–95). *Bifidobacterium* spp. can break down diet-derived glycans like xylan and host-derived carbohydrates like mucin (92, 93). The resulting products, such as sialic acid, glucose, 1,2-propanediol, acetate, and lactate, can be used by other microbes, including other *Bifidobacterium* spp., *Lactobacillus reuteri*, and *Eubacterium hallii (47, 96, 97*). This could influence the overall diversity and structure of the microbiome.

Mutualism is a symbiotic or bidirectional interaction where both species of bacteria benefit. Other studies have also defined similar interactions using terms like syntropy or synergy, and often refer to the exchange of metabolic products (47, 98, 99). Previous correlation analysis of integrated metagenomic and metabolomic data revealed a potential microbe-microbe interaction between probiotic *Faecalibacterium prausnitzii* and tumor-promoting *Peptostreptococcus stomatis* through mutualistic cross-feeding that could promote tumorigenesis (31). Understanding how metal availability affects microbial interactions and how metals mediate microbial signaling is key for understanding these interactions during gut inflammation. Under iron-limited conditions, *Bacteroides* spp. become dependent on their ability to capture siderophores like salmochelin and enterobactin from Enterobacteriaceae, including *Bacteroides thetaiotaomicron*, which is outcompeted during colitis if it is defective in salmochelin/enterobactin uptake systems (64, 100). During Environmental Enteric Dysfunction, a disorder contributing to child malnutrition and characterized by expanded Pseudomonadota, *Bacteroides* spp. improve carbohydrate access for *E. coli* through liberation of sialic acid, and *E. coli* improves iron acquisition for *Bacteroides* spp. through siderophore secretion and hemoglobin degradation (100, 101).

Additionally, the production of metabolic byproducts can influence bacteria that promote inflammation. Host-derived sialic acid from *Bacteroides* spp. can promote pathogen expansion in colitis (100, 102). This can fuel enteric bacteria including *E. coli* and others (102, 103). *S. enterica* serovar Typhimurium (*S. Tm*) and pathogenic *E. coli* use aspartate ammonia-lyase-dependent fumarate respiration for growth in the murine gut during inflammation that is fueled by microbiota-derived aspartate (104). Propionate production by *Bacteroides* spp. can mediate the colonization resistance of *S. Tm* (105). In the inflamed gut, *S. Tm* expands by metabolizing propionate through a mechanism that relies on nitrate-dependent anaerobic respiration to overcome colonization resistance (105).

SCFAs, such as acetate, propionate, and butyrate, are important metabolites for maintaining intestinal homeostasis by fueling colonocytes and generally lowering inflammation (25, 106–108). Low concentrations of SCFAs have been associated with gut inflammation, and patients with CD have lower concentrations of SCFAs than healthy individuals (86, 109, 110). This can be linked to the reduction of fiber-fermenting bacteria in the gut during dysbiosis that produce SCFAs but could also be impacted by AIEC (86, 110). AIEC has been shown to consume SCFAs better than other commensal *E. coli*, potentially leading to a decrease in SCFAs *in vivo* (25). Additionally, exposure to specific SCFAs has been shown to influence AIEC phenotypes and virulence. SCFAs butyrate and propionate promote increased AIEC adhesion and invasion of intestinal epithelial cells *in vitro* due to the upregulation of flagellar gene expression (25, 111). Propionate also promotes the virulence and persistence of AIEC by increasing survival in macrophages and upregulating genes involved in biofilm formation, stress response, metabolism, and others (112, 113). These changes in AIEC virulence are dependent on gut biogeography as pH and SCFA concentrations change along the GI tract (114). More work is needed to study the influence of gut metabolites like SCFAs on pro-inflammatory bacteria and their interactions.

Other positive interactions involve the degradation or production of mucin, the major protein component of mucus that lines and protects the intestines. As mucus is depleted during IBD due to degradation and reduced mucin secretion, interactions affecting mucin levels are key during inflammation. Pathogenic *E. coli* carries a fucose-sensing system that regulates virulence gene expression, and *B. thetaiotaomicron* can cleave fucose from mucin and enhance *E. coli* virulence (115). A beneficial interaction occurs between AIEC and the mucolytic symbiont *Akkermansia muciniphila* during periods of l-serine restriction (116). *Akkermansia* degrades the mucus layer, promoting the movement of AIEC closer to the epithelium where it can acquire L-serine and proliferate (116). An example of mutualism involves anti-inflammatory *F. prausnitzii* and *B. thetaiotaomicron*. To proliferate in germ-free rats, *F. prausnitzii* requires a preculture of *B. thetaiotaomicron* that reduces the oxido-reductive potential of the GI tract and enables *F. prausnitzii* colonization (117, 118). Additionally, *F. prausnitzii* produces acetate that *B. thetaiotaomicron* consumes in order to release butyrate, a compound that stimulates gut mucin production (117, 118). This mucin production leads to increased biofilm formation, which is favorable to the growth of these microbes. Other studies show increased growth and biofilm mass when gut microbial strains are grown in combinations of two to four species on mucin compared to individually, indicating synergistic interactions in multispecies biofilm formation (99).

More work is needed to further identify and probe these types of positive interactions. Some studies have conducted more correlative analyses among members of the microbiome to look for potential interactions. In our recent study, we did Spearman’s coefficient calculations to look for other members of the microbiota that were positively or negatively correlated with *Escherichia* and found that *Enterococcus* was positively correlated with *Escherichia* in the inflamed gut using *Il10^−/−^* mice (119). Another study has identified potential microbe-microbe interactions by developing a method to identify microbial modules or groups of taxa with similar abundance patterns in IBD (120). These modules have closer intragroup relationships and are thus more likely to have microbe-microbe interactions (120). Recent work using metagenomic data sets identified stably correlated genome pairs using co-abundance networks that could be used to identify a core microbiome signature to target for improved health (121).

## SIGNALING DURING MICROBIAL INTERACTIONS

The microbes in the GI tract undergo complex polymicrobial interactions through many signaling processes. Bacteria can receive signals through direct contact with other cells or through chemical signals in the form of metabolites or molecules packaged in extracellular vesicles (EVs) (122–124).

### Contact-dependent signaling

Contact-dependent interactions are essential for microbial community function and are key to coordinating metabolism and division of labor (125). These signaling mechanisms help the microbiota co-localize with their interacting partners and establish stable interactions, as spatial organization of the gut microbiota is important for establishing physical interaction (125, 126). Contact-dependent signaling includes contact-dependent inhibition (CDI), T6SSs (which have been described above), and nanotubes.

A CDI system was first discovered in *E. coli* and later in other Pseudomonadota and can be used for exerting inhibitory activity and kin recognition (125, 127, 128). CDI requires a specific receptor on the surface of the target cell to translocate different toxin effector domains with various modes of inhibition (129). Bacteria containing CDI systems also have immunity proteins that can neutralize the toxin and protect the producing cell (127, 129). CDI-mediated interactions occur between closely related bacteria and in some cross-species interactions (125, 130). For example, *Enterobacter cloacae* delivers effectors into diverse Enterobacteriaceae like *Escherichia*, *Klebsiella*, *Enterobacter*, and *Salmonella* species (125, 130). Thus, the composition of Enterobacteriaceae in the microbiome could be shaped by CDI-mediated interactions. In fact, it was found that CDI systems in *Burkholderia*, *Escherichia*, and *Pseudomonas* are essential for structuring microbial communities and can impact biofilm formation (131–134).

Nanotubes are conduits that bridge neighboring cells and allow the transfer of cytoplasmic molecules that can be cooperative or antagonistic (135–137). Nanotube-like structures have been reported in diverse commensal gut microbiota and reported to be produced by several enteric pathogens, including *Helicobacter pylori*, *Salmonella Typhimurium*, and *Vibrio* spp., to connect bacteria during biofilm formation and help attach to eukaryotic cells (125, 138, 139). Nanotube connections are induced to facilitate the movement of nutrients and can cause auxotrophic bacteria to promote overproduction of amino acids in donor cells by preventing feedback inhibition (136, 140). Bacteria can also communicate with more distantly located bacteria through the formation of elongated nanotubes (136, 141). The impact of nanotubes on the whole microbial community has not been explored; thus, more work is needed to understand the potential effect of nanotubes on community structure and host inflammation.

### Contact-independent signaling

There are numerous examples of small-molecule metabolites mediating microbe-microbe interactions. Many of the interactions described above were mediated through metabolite signaling. Key metabolites involved in signaling include SCFAs, products of mucin degradation, amino acids, and specialized metabolites like colibactin and siderophores. However, our microbiota produces thousands of unique small molecules and metabolites (142, 143). Much work has been done to better predict the chemical potential of the microbiome by identifying metabolic and BGCs from metagenomic DNA (66, 143, 144). Comparing metagenomes from fecal samples of individuals with IBD to healthy controls suggested that IBD was associated with the gain or loss of enzymes important for metabolic pathways, leading to changes in microbial metabolic profiles (145, 146). Using a multi-omics approach, putative mechanistic associations have been made between the IBD microbiome and its metabolites (147). This includes an increase in sphingolipids and bile acids, a decrease in triacylglycerols, and metagenomic changes suggesting adaptation to oxidative stress in the IBD microbiome (147). More work is needed to better understand the role of the gut metabolome in mediating microbial interactions and impacting disease.

EVs are small, membrane-bound particles released from almost all cell types into the extracellular space and can be used to transmit signals between cells (123, 148). EVs carry bioactive cargo including RNAs, DNA, proteins, carbohydrates, and lipids (149–151) and have been widely studied for their role in mediating host-microbe interactions. However, EV signaling between microbes, especially in the complexity of the gut microbiota, is less well-characterized. Work has been done to elucidate the roles of some EV signaling between microbes that involve both positive and competitive interactions. Various *Bacteroides* species produce EVs that carry polysaccharide-digesting enzymes that can be used by neighboring cells (152–155). Microbiota-derived EVs carrying glycoside hydrolases or polysaccharide lyases can break down polysaccharides to be consumed by all bacterial species, not just the enzyme producers (152, 154). These are public goods that benefit the whole microbial community (152). *Bacteroides* spp. can also produce EVs that carry cephalosporinases that degrade β-lactam antibiotics to protect the neighboring community (156). *E. coli* EVs can confer resistance to the antimicrobial peptide melittin in the producer *E. coli* and in *Pseudomonas aeruginosa* and *Acinetobacter radioresistens,* likely due to the presence of proteases on the EV surface (157). EVs can also serve as vehicles for horizontal gene transfer, especially for genes involved in antibiotic resistance, virulence, metabolism, and membrane synthesis (158–160). EVs can function as carriers for quorum sensing signals involved in coordinated phenotypes like biofilm formation and can also disseminate antimicrobial proteins and bacteriocins to kill competing microbes (123, 125, 161, 162).

The role of EV signaling in the pathogenesis of IBD is even less clear. Studies have focused on how EVs mediate host-microbe interactions in IBD, which have shown that bacterial EVs can modulate intestinal epithelial barrier integrity, mediate immune regulation, and modulate host cell metabolism (163). Like the microbiota as a whole, metagenomic analysis of the composition of EVs from fecal samples of patients with CD showed reduced microbial richness of DNA in EVs, especially altering the abundance of DNA from families and genera within Bacillota and Pseudomonadota (164). AIEC EVs have been shown to increase AIEC invasiveness through interactions between outer membrane protein A on AIEC EVs and endoplasmic reticulum stress response chaperone Gp96 on intestinal epithelial cells (165). More work is needed to study the impact of EV-mediated microbe-microbe interactions on inflammation and IBD progression.

## BIOFILMS

Biofilms on the gut mucosa have been linked to many health conditions including IBD, colon cancer, and chronic gut wounds (33, 34). In patients with IBD, 90–95% exhibit elevated concentrations of biofilms, in contrast to only 35% of healthy individuals (166). Biofilms are mono- or polymicrobial assemblages of bacteria that adhere to surfaces and are encased within a self-produced extracellular matrix that includes proteins, exopolysaccharides, and DNA (167). While there is debate about methods to measure and distinguish biofilms in the gut, it is generally defined as aggregates of bacteria encased in a matrix that adhere to food particles, mucus, or intestinal epithelia (32, 33). Mucosal biofilms are indicators for transitioning into a disease state with a compromised mucus layer facilitating colonization and biofilm formation (168). The outgrowth of biofilms, specifically mucosal pathogenic biofilms, may serve as an early indicator of IBD by differentiating the changes between a healthy and abnormal state in the gut microbiome. However, there is a need to distinguish adhesion, microcolonies, and true biofilm formation in the gut, as the understanding of mucosal biofilms in a healthy microbiota is limited (33). Biofilm communities have been shown to promote colon inflammation and tumor formation, and in particular, right-sided tumors have been highly correlated to polymicrobial biofilms (169–173). Biofilm-forming bacteria can be found primarily in the mucosa of the oropharynx and GI tract and occur in both CD and UC (166). Biofilms create a protective barrier against antimicrobial agents and immune responses, enhancing the survival and colonization of encased pathobionts during the initiation of stress pathways and contributing to recurring inflammation (34). Thus, understanding how bacterial interactions influence biofilm formation is important for determining best practices for treatment outcomes for IBD.

Additionally, biofilms associated with IBD are more virulent than those from healthy controls, as co-culture experiments with human intestinal epithelial cells demonstrated increased translocation of IBD-biofilm-forming bacteria (174). Pathobionts such as *Bacteroides fragilis* (*B. fragilis*), AIEC, and *Enterococcus faecalis* (*E. faecalis*) are commonly recognized for their high mucosal biofilm concentrations (175). AIEC can be found on the external surface of epithelial cells and in macrophages with an increased abundance in the mucosa compared to healthy individuals (176). AIEC has been shown to possess stronger biofilm formation compared to non-AIEC strains when present in the intestinal mucosa (36). The Type IV secretion system (T4SS) is an essential factor in the biofilm formation of these pathobionts by enhancing colonization on the epithelial surface through adhesion (177). The upregulation of T4SSs in CD-associated bacteria possibly contributes to disease progression by producing pili on external/internal surfaces, increasing bacterial DNA transfer and initiating biofilm formation both *in vivo* and *in vitro* (86). In support of this, Elhenawy et al. discovered that AIEC isolated from individuals diagnosed with CD needed T4SSs and the secretion of cellulose to form microcolonies on the epithelial surface (178). A T4SS mutant confirmed these findings as these samples displayed a scarcity of biofilm production compared to wild type (178). The production of the exopolysaccharide, cellulose, is an essential factor in *E. coli* biofilm formation and in AIEC-host interactions (179). In AIEC strain NC101, disrupted cellulose production reduced phagocytosis in iron-limiting conditions *in vitro* (180). Additionally, cellulose modulated macrophage pro-inflammatory responses based on iron availability and enhanced AIEC fitness and persistence in the gut, contributing to sustained immune activation (180). Development of these biofilms allows AIEC to colonize and persist in the GI tract, leading to positive feedback for the release of TNF-α from intracellular macrophages, possibly leading to the chronic inflammation seen in CD (181).

In addition to AIEC, the opportunistic pathogen *E. faecalis* also shows a high colonization rate in individuals with active CD (182). *E. faecalis* releases gelatinase and serine protease during infection to encourage colitis (175). This species of *Enterococcus* can form biofilms at various regions of the GI tract, contributing to its persistence during infection (183). *E. faecalis* thrives in a polymicrobial biofilm with *E. coli,* as interactions between these two microbes promote *E. coli* biofilm formation and growth due to the production of the metabolite, l-ornithine, released in response to infection (124). This amplifies siderophore production of *E. coli* in iron-limiting conditions caused by host-immune responses to create a synergistic relationship. These individual and polymicrobial biofilms exacerbate IBD by fostering microbial dysbiosis, disrupting the gut barrier, and inducing chronic immune activation. Their persistence makes inflammation difficult to resolve, contributing to the chronic and relapsing nature of the disease.

## DISCUSSION

**Fig 1:**
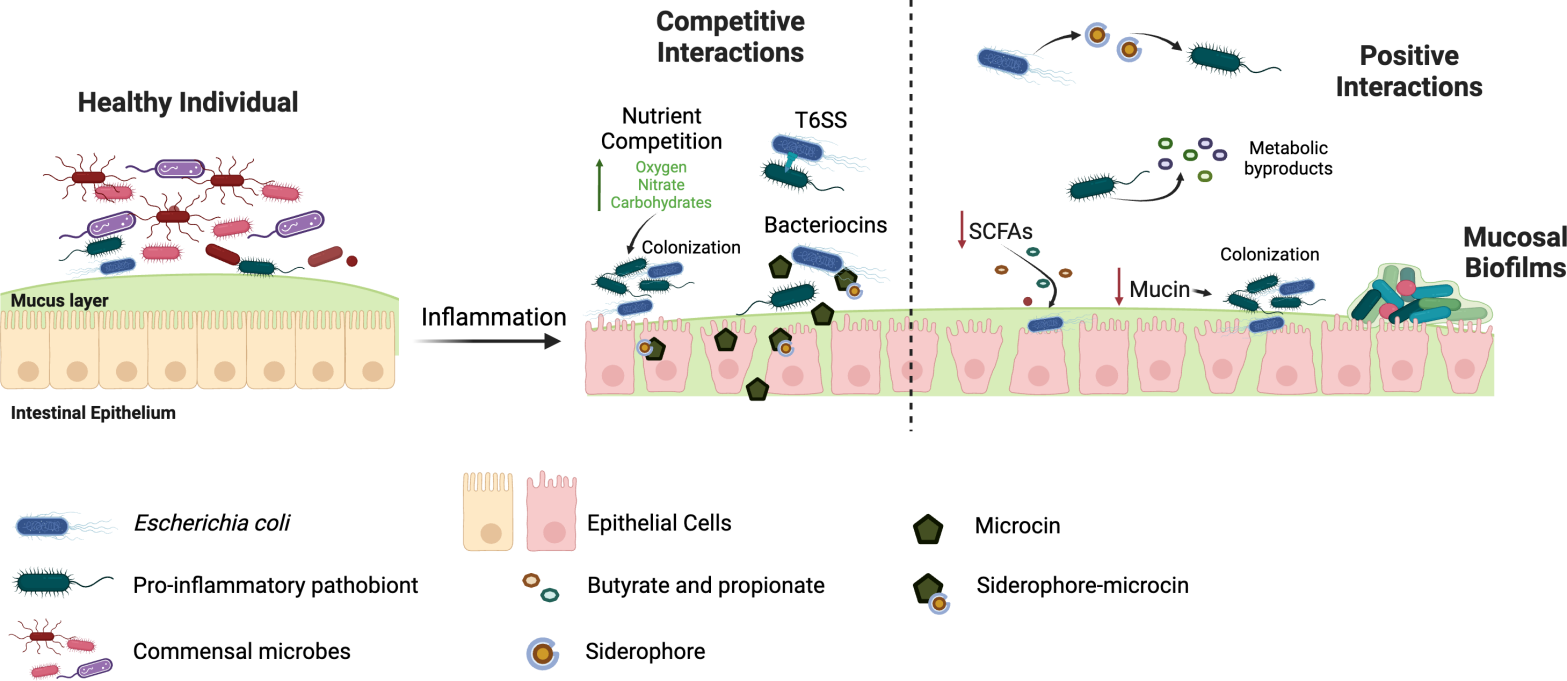
Microbe-microbe interactions that can influence microbiome dysbiosis and host inflammation. Competitive interactions are mediated by limited or altered nutrients in the inflamed gut, T6SSs, and specialized metabolites like bacteriocins. Positive interactions that benefit one or more bacteria involved include exchanging metabolic byproducts, taking up siderophores under iron limitation, and access to nutrients and increased biofilm formation due to mucin degradation.

During IBD, the environment of the GI tract changes due to chronic inflammation, which influences the composition and function of the gut microbiota. This dysbiosis within the microbiota and the crosstalk between host and microbiota influence the progression of IBD through various mechanisms (184). However, bacterial interactions are also pivotal in shaping the diversity and function of the gut microbiota (Fig. 1). There is a crucial gap in our understanding of microbe-microbe interactions within the GI tract, especially in how they influence inflammation and host health. It can also be unclear how to define interactions or even what constitutes a community in the context of the gut (28). Based on bulk sequencing data, it is difficult to determine if microbes are interacting within the approximately 6 m long GI tract (28). More work on understanding the spatial organization of the microbiota and examining the mucosal niche where most host-microbe interactions occur could help better define interacting bacterial partners important for disease. Biofilms in the gut are hard to define *in vivo* as standard practice has been to look at the microbial density of bacterial aggregates on food particles, mucus, or epithelia (32). Thus, further experiments are needed for studying mechanisms to better define biofilms in the gut (32). Recent technologies continue to improve our ability to image and study the spatial organization of the microbiota through single-cell RNA sequencing, spatial sequencing, imaging mass spectrometry, and improved imaging technologies (185–189). Microbial interactions could also be studied in complex microbiota using bacteriophages to deplete specific bacteria and determine the impact on the microbial community (190). These technologies should also provide more information about the actual function of microbes within their communities and more mechanistic insights into their interactions (28).

Another option to improve our understanding of the complex interactions between microbes and the influence of microbes on disease is to use more advanced computational models and machine learning. These are starting to be used more commonly to improve our understanding of microbial interactions and their role in disease (191). One example is using models to predict microbe-disease associations through novel approaches based on a dual-branch graph convolutional network module (192). As we use these modeling techniques, further experimental validation will be needed, thus making strategies for being able to genetically modify members of the gut microbiota even more imperative. New advances and technologies for genetically modifying gut bacteria are on the rise and will help us uncover the molecular mechanisms behind these microbe-microbe interactions (193). More recently, mathematical modeling has been used in concert with microbial bidirectional culturing to determine how gut commensals engage in the cross-feeding of metabolites, which could continue to be applied to study cross-feeding interactions among inflammatory microbes (194).

Future studies that integrate more of these multi-omics approaches could help uncover more key bacterial interactions and help with IBD treatment strategies (195). As we better understand microbial interactions, we can improve our development of biotherapeutics to treat IBD (196). This could include a more informed choice of probiotic use or building better bacterial consortia for therapeutic use (196). This knowledge is important as probiotics like *Lactobacillus plantarum* have been shown to alter the composition of the gut microbiome in models of colitis by increasing the ratio of Bacillota to Bacteroidota and in another study, raising the level of *Lactobacillus* (a member of the Bacillota phylum) and lowering the level of *Proteus* (an Enterobacteriaceae and member of Pseudomonadota) (197–199). Additionally, large-scale studies of microbiome evolution could help uncover how disease has shaped the gut microbial community, leading to the ability to identify strains and genes linked to inflammation and IBD (200). This work has been done to identify strain lineages strongly associated with IBD and likely adapted to IBD (200).

We can use our knowledge of microbe-microbe interactions for more targeted microbiota management for IBD. Bacterial EVs have been considered as a possible treatment for IBD (201). EVs from *Clostridium butyricum* reduced murine dextran sodium sulfate (DSS)-induced colitis by remodeling the composition of the microbiota and reducing the abundance of *E. coli* and *Shigella flexneri* (202). EVs derived from *L. plantarum* also improved dysbiosis of the gut microbiota and promoted microbial diversity in a colitis mouse model (203). EVs from *A. muciniphila* enhanced tight junction function and decreased the gut permeability of LPS-treated Caco-2 cells (204). *Roseburia intestinalis*-derived EVs increased the abundance of *Bifidobacterium* and improved inflammation and intestinal recovery in a DSS-induced colitis model (205). Another way to target specific strains in the microbiota is through bacteriophage therapy, which has shown some efficacy against AIEC and others (206–208). Finally, molecules can be used that specifically target bacterial features. For example, tungstate treatment is effective against Enterobacteriaceae during gut inflammation by selectively inhibiting molybdenum-cofactor-dependent microbial respiratory pathways (209). Use of these microbiota-targeted therapeutics highlights the importance of the microbiota in IBD and the role of microbe-microbe interactions in shaping the microbiota and impacting disease progression.
